# Measurement properties of instruments to measure the fatigue domain of vitality capacity in community-dwelling older people: an umbrella review of systematic reviews and meta-analysis

**DOI:** 10.1093/ageing/afad140

**Published:** 2023-10-30

**Authors:** Veerle Knoop, Emelyn Mathot, Francis Louter, David Beckwee, Christopher Mikton, Theresa Diaz, Jotheeswaran Amuthavalli Thiyagarajan, Ivan Bautmans

**Affiliations:** Gerontology Department, Vrije Universiteit Brussel, 1090 Brussels, Belgium; Frailty in Ageing (FRIA) Research Department, Vrije Universiteit Brussel, 1090 Brussels, Belgium; SOMT University of Physiotherapy, Amersfoort, The Netherlands; Gerontology Department, Vrije Universiteit Brussel, 1090 Brussels, Belgium; Frailty in Ageing (FRIA) Research Department, Vrije Universiteit Brussel, 1090 Brussels, Belgium; Gerontology Department, Vrije Universiteit Brussel, 1090 Brussels, Belgium; Gerontology Department, Vrije Universiteit Brussel, 1090 Brussels, Belgium; Frailty in Ageing (FRIA) Research Department, Vrije Universiteit Brussel, 1090 Brussels, Belgium; Demographic Change and Healthy Aging Unit, Social Determinants of Health, World Health Organization, Geneva, Switzerland; Epidemiology, Monitoring and Evaluation Units, Department of Maternal, Newborn, Child and Adolescent Health and Ageing, WHO HQ, Geneva, Switzerland; Ageing and Health Unit, Department of Maternal, Newborn, Child and Adolescent Health & Ageing, WHO HQ, Geneva, Switzerland; Gerontology Department, Vrije Universiteit Brussel, 1090 Brussels, Belgium; Frailty in Ageing (FRIA) Research Department, Vrije Universiteit Brussel, 1090 Brussels, Belgium; SOMT University of Physiotherapy, Amersfoort, The Netherlands; Department of Geriatrics, Universitair Ziekenhuis Brussel (UZ Brussel), 1090 Brussels, Belgium

**Keywords:** healthy ageing, intrinsic capacity, frailty, fatigue, energy, vitality capacity, aged, ageing, independent living

## Abstract

**Background:**

Vitality capacity (VC) is a key domain of intrinsic capacity (IC) and is the underlying biophysiological aspect of IC. Energy and metabolism (E&M) is one of the domains of VC. Fatigue is one of the main characteristics of E&M.

**Objective:**

The aims of this umbrella review are (i) to identify the available instruments suitable for measuring fatigue in community-dwelling older adults and (ii) to critically review the measurement properties of the identified instruments.

**Design:**

Umbrella review.

**Setting:**

Healthcare.

**Subjects:**

Community-dwelling older adults.

**Methods:**

PubMed and Web of Knowledge were systematically screened for systematic reviews and meta-analysis reporting on fatigue instruments resulting in 2,263 articles (last search 5 December 2022). The COSMIN checklist was used to appraise psychometric properties and the AMSTAR for assessing methodological quality. Data on fatigue instruments, construct, reference period, assessment method, validated population, reliability, validity, responsiveness and predictive validity on negative health outcomes were extracted.

**Results:**

10 systematic reviews and 1 meta-analysis were included in this study. 70 fatigue instruments were identified in the literature and 21 were originally designed for fatigue. The Fatigue Severity Scale (FSS), Pittsburgh Fatigability Scale (PFS) and Visual Analogue scale (VAS-F), Fatigue Impact Scale (FIS) and the Functional Assessment of Chronic Illness Therapy Fatigue (FACIT-F) presented good psychometric properties.

**Conclusions:**

The FSS, FIS, FACIT-F, PFS and the VAS-F presented good psychometric properties in various conditions. Therefore, these instruments could be used to quantify trajectories in the domain E&M in the context of VC in community-dwelling older adults.

## Key Points

- 21 different fatigue instruments were identified with moderate/good psychometric properties
- Self-perceived fatigue instruments were more represented than muscle fatigue instruments
- The FSS, FIS, FACIT-F, PFS and VAS-F can quantify fatigue in the context of vitality capacity
- Measuring fatigue as a predictor of vitality capacity can lead to more preventive interventions

## Introduction

The United Nations’ declaration of the Decade of Healthy Ageing for 2021–30 highlights the growing importance of addressing the challenges associated with ageing population. The World Health Organisation (WHO) has introduced the concept of ‘healthy ageing’ (HA), which focuses on maintaining functional ability and overall well-being in older age, rather than solely focusing on disease management [1]. A key concept in HA is intrinsic capacity (IC), referring to the physical and mental capacity an individual can draw on at any given time. WHO has proposed a framework for IC, including locomotive, psychological, sensorial, cognitive and vitality domains.

Vitality capacity (VC) has been suggested as a core domain of IC, constituting the underlying physiological state [2]. However, a WHO regulated definition of VC was lacking in the literature. To advance the measurement and monitoring of VC the WHO had organised an expert meeting with 20 international experts to discuss and clarify the attributes of VC and to develop a conceptual definition. This has led to a new WHO definition, defining VC as ‘a physiological state (due to normal or accelerated biological ageing processes) resulting from the interaction between multiple physiological systems, reflected in (the level of) energy and metabolism, neuromuscular function, and immune and stress response functions of the body’ [3]. Energy and metabolism (E&M) is one of the domains of VC in the new definition.

The experts indicated their top biomarkers for each of the domains of VC and identified self-perceived *fatigue and muscle fatigability* alongside other biomarkers as important for quantifying E&M [3]. The experts indicated fatigue as a feasible to quantify, feasible to measure, useful for monitoring and implementable biomarker that matched with all the criteria needed for a biomarker measuring E&M in the context of VC [3]. Fatigue could be a state of energy deficiency and is influenced by several biological changes that are related to ageing such as decline in mitochondrion function [4, 5] and low-grade inflammation [6], which makes that fatigue can be linked to accelerated ageing [7]. Fatigue is affected by biological ageing and higher levels of physiological systems, indicating that fatigue is one of the biomarkers of VC [2, 3]. Fatigue can characterise the absence of energy from a physiological perspective [8] and can be a response to conserve energy [9], and is therefore seen as a biomarker for E&M.

Fatigue is a common complaint among older adults and is one of the main reasons to consult a general practitioner [10]. Fatigue is one of the identified symptoms by patients who recovered from COVID-19 and is the most common long-term effect after a COVID-19 infection [11]. However, fatigue remains complex due to its large array of underlying mechanisms and related constructs. Fatigue is defined as follows: *“Fatigue is a state usually associated with a weakening or depletion of one”s physical and/or mental resources, ranging from a general state of lethargy to a specific, work-induced burning sensation within one’s muscles’* [12]. Two different constructs of fatigue can be distinguished [13]: on the one hand, self-perceived fatigue; and on the other hand, muscle fatigability. Self-perceived fatigue reflects a perception-based sensation expected to normally occur at certain times of the day and after activities [13]. Muscle fatigue leads to the inability to continue functioning at a normal level of activity and muscle fatigability refers to the susceptibility of the muscle for muscle fatigue and reduced tolerance for muscular work [14]. Self-perceived fatigue is often measured through self-reported questionnaires while muscle fatigability instruments measure objectively the point of muscle failure [13]. Many fatigue instruments have been validated among older persons with chronic conditions. Higher levels of fatigue increase the risk for negative healthy outcomes such as hospitalisation, diseases and mortality [15] and are related to frailty [16] in non-diseased older adults. Therefore, it is critical to accurately assess fatigue in community-dwelling older adults.

The United Nations’ Decade of Healthy Ageing (2021–23) calls for strengthening measurement for monitoring health and well-being of older people, including the measurement of VC [17]. There is a lack of systematic reviews for validated instruments for assessing accurately fatigue among community-dwelling older adults. Therefore, the aims of this umbrella review are (i) to identify available instruments suitable for measuring fatigue in community-dwelling older adults and (ii) to critically review the measurement properties of the identified instruments.

## Methodology

### Study protocol and registration

The Preferred Reporting Items for Systematic Reviews and Meta-Analyses (PRISMA) statement guided the methodology and reporting of this systematic review [18]. The protocol of this systematic review was registered in PROSPERO (CRD42023390383).

### Search strategy

Databases PubMed and Web of Knowledge were systematically screened (last search 5 December 2022). For this review, the following PICO question was used: ‘Which instruments (I) for measuring fatigue (self-perceived/muscle fatigability) (O) in older adults (P) exist’? The used keywords are shown in Box 1.

Box 1 Used keywords used for PubMed and Web of Knowledge

**PubMed:** (‘Fatigue’[Mesh]) OR (‘Muscle Fatigue’ [Mesh]) OR (fatigability)) OR (tiredness)) OR (exhaustion)) OR (muscle endurance)) AND (‘Prognosis’ [Mesh]) OR (‘Risk’[Mesh]) OR (assessment) OR (outcome)) OR (predictor) OR (psychometrics))) OR (‘Reproducibility of Results’[Mesh]) AND (‘Systematic Review’ [Publication Type]) OR (‘Cohort Studies’[Mesh]) OR (‘Prospective Studies’[Mesh]) NOT (‘Multiple Sclerosis’[Mesh]) NOT (‘Hospitalisation’[Mesh] NOT (Hospitalised) NOT (cancer) NOT (stroke) NOT (Covid-19) NOT (HIV) with the filter ‘Systematic Review’ **Web of Knowledge:** (Topic = Fatigability OR Tiredness OR Exhaustion OR Muscle Fatigue OR Muscle Endurance) AND (Topic = Prognosis OR Risk OR Predictor OR Assessment OR Outcome OR Predictor OR Psychometrics OR Reproducibility of Results OR validity OR reliability) AND (Topic = Systematic reviews) NOT (Cancer NOT Multiple Sclerosis NOT Fibromyalgia) for Web of Science.

### Selection criteria

Studies were eligible if they investigated fatigue or fatigue instruments in adults (no age limit to avoid missing relevant fatigue scales). Other inclusion criteria were inclusion of fatigue instruments as the focus, written in English or Dutch and systematic reviews or meta-analyses. Studies focusing *only* on people with specific disease groups were excluded since the presence of fatigue in those groups is more affected by the nature of the disease than age-related changes. Moreover, this review is not focusing on disease-specific instruments to measure fatigue. It can be difficult to ascribe fatigue to a single disease or cause; for this reason, we excluded articles with specific health conditions. Articles inducing fatigue or fatigue protocols were excluded. Two authors performed the screening process independently, blinded to each other’s results. Disagreement was resolved by a third author through discussion and consensus. Articles were firstly screened based on title and abstract. Subsequently, full texts were screened (Appendix S1).

### Data collection process and data extraction

A five-step approach was followed (Appendix S2). First, all fatigue instruments reported in the systematic reviews and meta-analyses were extracted and divided into self-perceived fatigue and muscle fatigability instruments. The following data were extracted: study, population, instrument, presence of psychometric properties and predictive validity. Second, for all the selected fatigue instruments, the scoring system, number of items, validated population (children/adults/older adults) and the original aim of the instrument was reported. Third, fatigue instruments primarily designed for fatigue and validated in older adults were selected; consequently, only instruments designed for fatigue and validated in older adults were included. To verify whether the instruments was designed for fatigue, we used the following definition: *“Fatigue is a state usually associated with a weakening or depletion of one”s physical and/or mental resources, ranging from a general state of lethargy to a specific, work-induced burning sensation within one’s muscles’* [12]. Fourth, the selected instruments were assessed for psychometric properties and predictive validity on negative health outcomes and longevity. Finally, the remaining fatigue instruments were assessed dichotomously (yes/no and unclear) by the following criteria: (i) feasibility to quantify biomarkers or proxy biomarkers, (ii) feasibility to measure or collect in low-resource settings, (iii) usefulness and informativeness for monitoring, (iv) distinctiveness of instrument, (v) acceptability regarding cost and resource demand, (vi) availability and no ethical concerns, (vii) implementability and (viii) robustness regarding psychometric properties.

### Measurement properties and quality assessment

The internal validity of the included reviews were assessed by two independent reviewers using the ‘measurement tool to assess the methodological quality of systematic reviews’ (AMSTAR) [19]. The COSMIN checklist was used to assess the psychometric properties, including validity (criterion, content, structural, predictive, cross-cultural), internal consistency, measurement invariance, reliability (test–retest) and hypothesis testing for construct validity [20]. Also, the COSMIN guidelines were used to evaluate the measurement properties of each scale in terms of adequate (+), inadequate (−) or indeterminate (?), or inconsistent (±) based on the study design and methodology. The interpretability is not considered to be a psychometric property under the COSMIN framework and is therefore not described in this review.

## Results

### Search results and quality assessment

**Figure 1:**
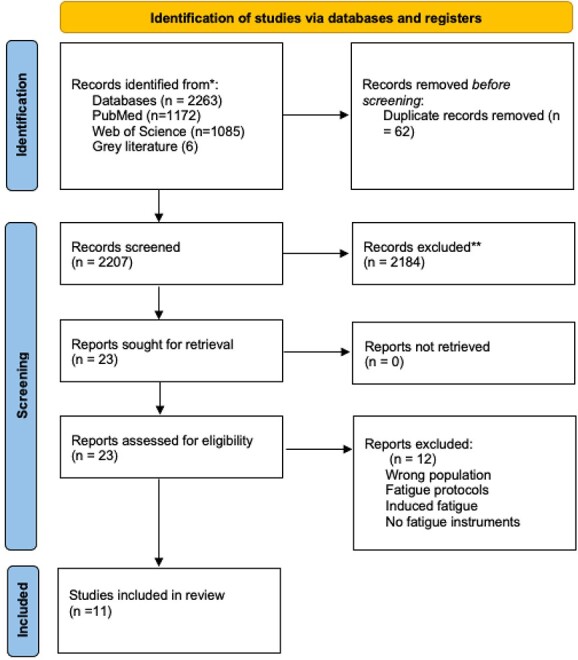
PRISMA flow diagram for identification of studies.

This review included 1 meta-analysis [15] and 10 systematic reviews [16, 21–29] (Table 1 and Figure 1) published between 2006 and 2022. The AMSTAR checklist indicated moderate methodological quality of the quality of the included reviews. The studies’ characteristics were well reported in almost all studies; only two studies assessed the likelihood for publication bias [15, 23]. All studies performed a robust selection process and data extraction. An overview of the applied checklist and the results per study can be found in the Appendix S3.

**Table 1:** Study characteristics

Author (year)	Population, age, gender, countries	Identified fatigue instruments	Muscle fatigability	Self-perceived fatigue	Psychometric properties published	Predictive validity on negative health outcomes
Díaz-García, González-Ponce [21] (2021)	Age: range 13–86 years Participants: *n* = 16,147 Gender: males and females Conditions: no comorbidities, cancer, depression brain injury, multiple sclerosis, Parkinson and stroke Countries: not specified	Chalder Fatigue Questionnaire (Chalder) Mental Fatigue Scale (MFS) Chronic Fatigue Questionnaire (CFS) Dutch Multifactor Fatigue Scale (DMFS) European Organisation for the Research and Treatment of Cancer quality of life questionnaire (EORTC QLQ-C3) Fatigue Assessment Scale (FAS) Functional Status Questionnaire (FSQ) Multidimensional Fatigue Inventory (MFI) Pittsburgh Fatigability Scale (PFS) Situational Fatigue Scale (SFS) State–Trait Inventory for Cognitive Fatigue (STI-CF)	No No No No No No No No No No No	Yes Yes Yes Yes Yes Yes Yes Yes Yes Yes Yes	No	No
Donovan, Stein [22] (2015)	Age: range 21.2–95.8 years Participants: *n* = 8,091 Gender: males and females Conditions; no comorbidities, cancer, insomnia, headache, cancer, heart disease, osteoarthritis, thrombosis, chronic fatigue syndrome, hypertension Countries: not specified	Multidimensional Fatigue Symptom Inventory–Short Form (MFI-SF)	No	Yes	Yes	No
Frestad and Prescott [23] (2017)	Age: range 18–93 years Participants: *n* = 53,168 Gender: males and females Conditions: no comorbidities and chronic heart failure Countries: The Netherlands, USA, Denmark, Sweden, Venezuela, Switzerland	Maastricht questionnaire (MQ)	No	Yes	No	Yes
Van Geel, Moumdjian [24] (2020)	Age: range 10–97 years Participants: *n* = 2,636 Gender: males and females Different conditions: no comorbidities, atrophy, osteoarthritis, stroke, interstitial lung disease, myasthenia gravis Countries: not specified	400 m walk Timed 500 m walking test 6MWT 10 meter walk 12 min walk 100 min walk Treadmill walk	Yes Yes Yes Yes Yes Yes Yes	No No No No No No No	Yes	No
Pana, Sourtzi [25] (2021)	Age: range 55–106 years, mean 73.68 Participants: *n* = 59,852 Gender: males and females Conditions: no comorbidities Countries: Sweden, USA, Brazil, Japan, Australia, UK, China, Spain, France	Center for Epidemiologic Studies–Depression Scale (CES-D) Short Form 36 Vitality subscale score (SF-36) General question (subjective) Short Form (SF-36) (3 items) InterRAI (fatigue domain) Japanese version of the SF 36 subscale Vitality Kihon checklist (1 item) Fatigue Severity Scale (FSS) Visual Analogue Scale Fatigue (VAS-F)	No No No No No No No No No	Yes Yes Yes Yes Yes Yes Yes Yes Yes	No	Yes
Knoop, Costenoble [16] (2019)	Age: ≥65 years Participants: not reported Gender: males and females Conditions: no comorbidities Countries: not reported	Center for Epidemiologic Studies–Depression Scale (CES-D) General question (subjective) SF-36 Vitality subscale score (SF-36) Geriatric Depression Scale (GDS) 5 times sit to stand test 30 seconds chair stand test (30s CST) European Organisation for the Research and Treatment of Cancer quality of life questionnaire (EORTC QLQ-C3) Beck Depression Inventory (BDI) Kessler Psychological Distress Scale (K10) Patient Health Questionnaire (PHQ) 12-Item Short Form Health survey (12-SF) Upper Extremity Exhaustion	No No No No Yes Yes No No No No	Yes Yes Yes Yes No No Yes Yes Yes Yes	No	No
Knoop, Cloots [15] (2021)	Age: range 20–98 years Participants: *n* = 152,711 Gender: males and females Conditions: no comorbidities Countries: not reported	Mobility Tiredness Scale (MOB-T) Lower Limb Tiredness Scale (LIMB-T) Generic question (subjective) SF-36 Vitality (1 item) Borg Rating of Perceived Fatigue Scale (BORG) Pittsburgh Fatigability Scale (PFS) SF-36 Vitality subscale (SF-36) Maastricht questionnaire (MQ) Fatigue resistance test (FR) GripWork (GW)	No No No No No No No No Yes Yes	Yes Yes Yes Yes Yes Yes Yes Yes No No	No	No
Mota and Pimenta [26] (2006)	Age: not specified Participants: *n* = 7,432 Gender: males and females Conditions: no comorbidities, cancer, chronic fatigue syndrome, multiple sclerosis Countries: USA, The Netherlands, UK, Canada, Australia, Japan	Fatigue Severity Scale (FSS) Visual Analogue Scale fatigue (VAS-F) Fatigue Assessment Instrument (FAI) Brief Mental Fatigue Questionnaire (BMFQ) Chalder Fatigue Scale (CFS) Fatigue Impact Scale (FIS) Multidimensional Fatigue Inventory-20 (MFI-20) Fatigue Symptom Inventory (FSI) Dutch Fatigue Scale (DUFS) Dutch Exertion Fatigue Scale (DEFS) Piper Scale (PIPER) Schwartz Cancer Fatigue Scale (SCFS) Brief Fatigue Inventory (BFI) Cancer Fatigue Scale (CaFS) Schedule of Fatigue and Anergia (SOFA) Cancer Related Fatigue Distress Scale (CRFDS) Fatigue Impact Scale for Daily Administration (FIS-D)	No No No No No No No No No No No No No No No No No	Yes Yes Yes Yes Yes Yes Yes Yes Yes Yes Yes Yes Yes Yes Yes Yes Yes	Yes	No
Nordin, Taft [27] (2016)	Age: >18 years Participants: *n* = 17,435 Gender: not reported Conditions: no comorbidities, cancer, multiple sclerosis, rheumatoid arthritis, Sjögren syndrome, psoriatic arthritis, spondyloarthropathy Counties: not specified	Chalder Fatigue Scale (CFQ) Chronic Heart Failure Questionnaire, Fatigue subscale (CHQ) Chronic Respiratory Questionnaire, Fatigue subscale (CRQ) Edmonton Symptom Assessment System (ESAS) European Organisation for the Research and Treatment of Cancer quality of life questionnaire (EORTC QLQ-C3) FACIT-F Fatigue Assessment scale (FAS) Fatigue Associated with Depression Questionnaire (FasD) Fatigue Impact Scale (FIS) Fatigue Severity Scale (FSS) Multidimensional Assessment of Fatigue (MAF) Multidimensional Fatigue Inventory-20 (MFI-20) Neurological Fatigue Index for multiple sclerosis (NFI-MS) Perform Questionnaire (PQ) Profile of Mood states-Fatigue (POMS) PROMIS-Fatigue Quality of Life Inventory in Epilepsy (QOLIE) Sleep Impact Scale (SCFS) Trial Outcome Index-Fatigue (TOI-F) Visual Analogue Scale Fatigue (VAS-F)	No No No No No No No No No No No No No No No No No No No No	Yes Yes Yes Yes Yes Yes Yes Yes Yes Yes Yes Yes Yes Yes Yes Yes Yes Yes Yes Yes	Yes	No
Su, Cochrane [28] (2022)	Age: >65 years Participants: not reported Gender: not reported Conditions: no comorbidities Countries: USA, Europe, Asia, other places	Pittsburgh Fatigability Scale (PFS) Fatigue Severity Scale (FSS) The Chinese MFI Center for Epidemiologic Studies–Depression Scale (CES-D) SF-36 Vitality subscale (SF-36) Mobility Tiredness Scale (MOB-T) State–Trait Inventory for Cognitive Fatigue (STI-CF) Tiredness after 6 min walking test Patient-Reported Outcomes Measurement Information System (PROMIS) Brief Fatigue Inventory (BFI) Turkish version of Checklist Individual Strength (CIS-T) FACIT-F Multidimensional Fatigue Inventory-20 (MFI-20) SF-12 questionnaire (12-SF) Visual Analogue Scale fatigue (VAS-F) Fatigue Assessment scale (FAS) Eight State Questionnaire (8SQ) Cohen–Hoberman Inventory of Physical Symptoms (CHIPS) Piper Fatigue Scale (PIPER)	No No No No No No No Yes No No No No No No No No No No No No	Yes Yes Yes Yes Yes Yes Yes No Yes Yes Yes Yes Yes Yes Yes Yes Yes Yes Yes Yes	Yes	No
Yu, Lee [29] (2010)	Age range: 65–100 years Participants: *n* = 5,781 Gender: males and females Conditions: no comorbidities, depression, Parkinson, Alzheimer, cancer, cardiovascular disease Countries: Denmark, USA, Australia, Belgium	Mobility Tiredness Scale (MOB-T) Lower Limb Tiredness Scale (LIMB-T) Short Form 36 (SF-36) Chronic Fatigue Questionnaire (CFS) Modified Piper Fatigue Scale (M-PIPER) Fatigue scale of the Eight State Questionnaire Six somatic symptoms from GHQ (8SQ) Visual Analogue Scale for fatigue (VAS-F)	No No No No No No No No	Yes Yes Yes Yes Yes Yes Yes Yes	No	Yes

*n* = number

### Characteristics of included studies and instruments

The included studies reported on 323,253 participants; two studies did not report the number of participants [16, 28]. In most studies, the participants consisted of both genders (not reported in five reviews [16, 24, 27–29]) and the age ranged between 10 and 100 years; the age was not reported in two studies [26, 27]. Most articles included community-dwelling or ‘older adults without chronic conditions’; however, some other articles also included older adults with chronic conditions [21, 24, 26, 29] and for one article the population was not specified [27].

In total, 70 different fatigue instruments were reported of which 58 focused on *self-perceived* fatigue and 12 on *muscle* fatigability (Table 2). Out of the self-perceived fatigue instruments, the SF-36 [30] was the most reported (*n* = 6), using either the full SF-36 [29], the vitality subscale [15, 16, 25, 28], three questions [25] or only one item from the SF-36 [20]. The FSS [31], VAS-F [32] and MFI-20 were reported five times. Only 12 fatigue assessments were reported for muscle fatigability. Different protocols of walking-related performance fatigability assessments were included, including the 6-minute walking test, 400-meter walk test, 10-meter walk test, 12-minute walking test, 100-meter walking test and treadmillwalk.

Table 3 shows 21 instruments that were initially designed for evaluating fatigue. Half of the instruments are multidimensional scales and 11 scales were unidimensional (BFI, FAS, FSS, MOB-T, PIPER, DUFS, DEFS, FIS, 12-minute walk test, FR and GW), i.e. investigating only one fatigue construct. The most common fatigue construct used in these instruments were general, physical and mental fatigue. Three instruments (12-minute walk test, FR and GW) were measuring solely physical fatigue through an objective method while the other instruments focused more on self-perceived fatigue and were all self-assessment scales. All instruments measured acute fatigue and the reference periods ranged between ‘present’, ‘usually’ and ‘in the past 2 weeks’ (except CFQ ‘past month’). Chronic fatigue was not assessed. Most instruments were initially validated in older persons with diseases; however, all the fatigue instruments have also been validated in general populations.

**Table 2:** Identified fatigue instruments and constructs

Fatigue instruments	N reported in the studies	Initially designed for	Validated population	Designed for fatigue	Scoring system	Number of items
**Self-perceived fatigue**						
MQ^(Appels et al., 1987)^	1	Symptoms of adaption to prolonged distress—vital exhaustion	Adults/older adults	NO	4 Quartiles ranging from low to high vital exhaustion	17–37 items
CESD^(Radloff, 1991)^	3	Depressive symptoms	Children/adults/older adults	NO	Cut off > 16 points	20 items
SF-36 Vitality subscale score^(Ware & Gandek, 1998)^	8	Vitality subscale: general measure of energy/fatigue	Adults/older adults	NO	5-point Likert scale (1 = all the time, 5 = none of the time) Lower scores indicate greater fatigue (0–100)	4 items
InterRAI (fatigue domain)^(Hogeveen et al., 2017)^	1	Physical, psychological and cognitive domains	Older adults	NO	4-point Likert scale	1 item
Kihon Checklist^(Arai, 2021)^	1	Frailty	Older adults	NO	Dichotomy	25 items (1 fatigue item)
FSS^(Krupp et al., 1989)^	5	Fatigue	Adults/older adults	YES	7-point Likert scale Range: Range (9–63) The higher the score the higher fatigue	9 items
VAS-F^(Lee et al., 1991)^	5	Fatigue	Adults/older adults	YES	Range: (0–180) The higher the score the higher fatigue	18 Items
GDS^(Yesavage et al., 1982)^	1	Depression	Older adults	NO	Fatigue item dichotomy	8 items 15 items 30 items 1 fatigue item
EORTC^(Aaronson et al., 1993)^	3	Quality of life	Adults/older adults	NO	Higher score indicating a better HRQoL 1: not at all, 2: a little, 3: quite a bit, 4: very much range (1–4)	30 items 1 fatigue item
BDI^(Beck et al., 1961)^	1	Depressive symptoms	Children/adults/older adults	NO	>13 points risk for depression Range of 3 ordinal	21 items (1 fatigue item)
K10^(Kessler et al., 2003)^	1	Depressive symptoms	Children/adults	NO	1: none of the time, 2: a little of the time, 3: some of the time, 4: most of the time, 5: all of the time	10 items (1 fatigue item)
PHQ^(Kroenke et al., 2001)^	1	Depressive symptoms	Adults/older adults	NO	0: not at all, 1: several days, 2: more than half of the days, 3: nearly every day	9 items (1 fatigue item)
12-SF^(Ware et al., 1996)^	2	Quality of life	Adults/older adults	NO	6 point Likert scale	12 items (1 fatigue item)
MOB-T^(Avlund et al., 1993)^	3	Mobility related fatigue	Older adults	YES	Dichotomous	6 items
LIMB-T^(Avlund et al., 1993)^	2	ADL related tiredness	Older adults	YES	Dichotomous	6 items
BORG^(Borg, 1990)^	1	Subjective experience of physical activity intensity	Adults/older adults	NO	From low to extremely heavy	1 item
PFS^(Glynn et al., 2015)^	3	Physical& mental fatigability	Older adults	YES	5-Points likert scale higher score = greater fatigue	40 items
MFI-20^(Smets et al., 1995)^	5	General fatigue, mental fatigue, physical fatigue, motivational fatigue, activity related fatigue	Adults/older adults	YES	5-point likert scale higher score = greater fatigue	20 items
STI-CF^(Shuman-Paretsky et al., 2017)^	2	Cognitive fatigue	Older adults	NO	11-point scale	32 items
CIS-T^(Vercoulen et al., 1994)^	1	Fatigue & behavior (General, concentration, motivation, activity)	Adults/older adults	YES	7-point Likert scale higher score = greater fatigue	20 items
FACIT-F^(Yellen et al., 1997)^	2	Fatigue & Quality of life	Adults/older adults	YES	5-point Likert scale higher score = lower fatigue	13 items
8SQ^(Boyle, 1991)^	2	Emotion & mood	Children/adults	NO	Not reported	8 items
CHIPS^(Cohen & Hoberman, 1983)^	1	Physical symptoms	Adults/older adults	NO	5-point Likert scale higher score = greater fatigue	33 items
Piper^(Piper et al., 1998)^	3	Tiredness	Adults/older adults	YES	10-point Likert scale + open questions)	22 items
CFQ^(Nijs et al., 2005)^	1	Activity and participation	Adults/older adults	NO	4-point Likert scale	26 items

*(Continued)*

**Table 2:** Continued

**Fatigue instruments**	**N reported in the studies**	**Initially designed for**	**Validated population**	**Designed for fatigue**	**Scoring system**	**Number of items**
GHQ^(Goldberg & Hillier, 1979)^	1	Psychologic disorders	Adults/older adults	NO	Several versions 4-point Likert scale	30–28-12 items
PROMIS^(Gershon et al., 2010)^	1	Depression, fatigue, pain, emotional support	Children/adults/older adults	NO	5-point Likert scale	563 items
MFI-SF^(Stein et al., 1998)^	1	General, physical, emotional, mental, and vigor	Adults/older adults	Yes	5-point Likert scale higher score = greater fatigue	30 items
BFI^(Mendoza et al., 1999)^	2	General fatigue	Adults/older adults	Yes	5-point Likert scale ≥ higher score = greater fatigue	9 items
FAS^(Michielsen et al., 2003)^	3	General fatigue	Adults/older adults	Yes	5-point Likert scale ≥ 22 severe fatigue	10 items
FIS^(Fisk et al., 1994)^	2	Physical fatigue, cognitive fatigue	Adults/older adults	Yes	5-point Likert scale Higher score = greater fatigue	40 items
Chalder^(Chalder et al., 1993)^	2	Fatigue	Adults/older adults	Yes	5-point Likert scale Higher score = greater fatigue	11 Items
MFS^(Johansson & Rönnbäck, 2014)^	1	Concentration and memory after mental activities	Adults	No	7-point Likert scale Higher score = greater fatigue	14 items
DMFS^(Visser-Keizer et al., 2015)^	1	Chronic fatigue after brain injury	Adults	No	5-point Likert scale Higher score = greater fatigue	30 items
FSQ^(Jette et al., 1986)^	1	Difficulties in ADL	Adults/older adults	NO	4-point Likert scale Higher score = greater fatigue	10 items
SFS^(Yang & Wu, 2005)^	1	Fatigue in specific activities of daily life in working population	Adults	Yes but not relevant for older adults	6-point Likert scale Higher score = greater fatigue	13 items
FAI^(Schwartz et al., 1993)^	1	Fatigue	Adults/older adults	Yes	7-point Likert scale Higher score = greater fatigue	29 items
BMFQ^(Bentall et al., 1993)^	1	Mental fatigue symptoms	Adults/older adults	No	5-point Likert scale Higher score = greater fatigue	9 items
FSI^(Hann et al., 1998)^	1	Intensity and duration of fatigue and its impact on quality of life	Adults	Yes	11-point Likert scale Higher score = greater fatigue	13 items
DUFS^(Tiesinga et al., 1998)^	1	Fatigue	Adults/older adults	Yes	Dichotomous	9 items
DEFS^(Tiesinga et al., 1998)^	1	fatigue	Adults/older adults	Yes	5-point Likert scale	9 items
CaFS^(Okuyama et al., 2000)^	1	Cancer related fatigue and quality of life	Adults/older adults	No	5-point Likert scale	15 items
SCFS^(Schwartz, 1998)^	4	Cancer related fatigue and quality of life	Adults/older adults	No	5-point Likert scale Higher score = greater fatigue	28 items
SOFA^(Hadzi-Pavlovic et al., 2000)^	1	Fatigue in complex medical syndromes	Adults	No	4-point Likert scale Higher score = greater fatigue	10 items
CRFDS^(Holley, 2000)^	1	Cancer-related fatigue and Quality of Life	Adults	No	Numeric scale (0–10)	20 items
FIS-D^(Fisk & Doble, 2002)^	2	Fatigue in daily life	Adults/older adults	Yes	5-point Likert scale Higher score = greater fatigue	8 items
CHQ^(Guyatt et al., 1989)^	1	Health status, dyspnoea, fatigue, emotional function	Adults	No	7-point Likert scale Higher score = quality of Life	16 items
CRQ^(Guyatt et al., 1989)^	1	Quality of Life	Adults/older adults	No	7-point Likert scale Higher score = Quality of life	20 items
ESAS^(Chang et al., 2000)^	1	Cancer Symptoms	Adults/older adults	No	Numeric scale (0–10)	9 items
FasD^(Matza et al., 2011)^	1	Depression-related fatigue	Adults	No	4-points Likert scale Higher score = greater fatigue	16 items
MAF^(Belza, 1995)^	2	Fatigue	Adults/older adults	Yes	Numeric scale (0–10) Higher score = greater fatigue	15 items
NFI-MS^(Mills et al., 2010)^	1	Multiple Sclerosis related fatigue	Adults	No	4-point Likert scale	12 items
PQ^(Baró et al., 2009)^	1	Cancer related fatigue	Adults/older adults	No	5-point Likert scale Higher score = greater fatigue	12 items
POMS^(McNair, 1992)^	1	Mood state	Adults/older adults	No	5-point Likert scale Higher score = greater fatigue	28 items
PROMIS-F^(Garcia et al., 2007)^	2	Tiredness	Children/adults/older adults	Yes	5-point Likert scale Higher score = greater fatigue	7 items
QOLIE^(Cramer et al., 1998)^	1	Disabilities in daily life	Children/adults	No	6-point Likert scale Higher score = greater fatigue	31 items

*(Continued)*

**Table 2:** Continued

**Fatigue instruments**	**N reported in the studies**	**Initially designed for**	**Validated population**	**Designed for fatigue**	**Scoring system**	**Number of items**
SIS^(Crawford, 2007)^	1	Impact of insomnia on quality of life	Adults	No	Higher score = greater fatigue	35
TOI-F^(Yellen et al., 1997)^	1	Anemia and fatigue	Adults/older adults	No	Higher score = greater fatigue	27
**Muscle fatigue**						
5CST^(Csuka & McCarty, 1985)^	1	Functional lower extremity strength	Adults/older adults	NO	Time	Scale
30sSCT^(Csuka & McCarty, 1985)^	1	Leg strength and endurance	Adults/older adults	NO	Number or times	Scale
UEE^(Toosizadeh et al., 2016)^	1	Injury and fatigue	Adults/older adults	NO	Electromyography (EMG)	Scale
6MWT^(Engelhard et al., 2016)^	2	Aerobic capacity and endurance	Adults/older adults	NO	Distance	Scale
FR^(Bautmans & Mets, 2005)^	1	Muscle fatigue	Adults/older adults	YES	Time	Scale
GW^(De Dobbeleer et al., 2019)^	1	Muscle fatigue	Adults/older adults	YES		Scale
400 M walk^(Simonsick et al., 2014)^	1	Walking-related performance fatigability	Older adults	NO	time	Scale
500 m timed walk test^(Schwid et al., 1999)^	1	Walking-related performance fatigability Ambulatory fatigability	Adults/older adults	NO	Time	Scale
10-meter walk^(Schnelle et al., 2012)^	1	Performance fatigability severity	Older adults	NO	Distance	Scale
12 min walk^(Goldman et al., 2008)^	1	Walking-related performance fatigability	Adults/older adults	YES	Distance	Scale
100 min walk^(Lester & Zhang, 2010)^	1	Walking-related performance fatigability	Adults/older adults	NO	Distance	Scale
Treadmill walk^(Sehle et al., 2014)^	1	Walking-related performance fatigability	Adults/older adults	NO	Combined with the BORG scale Time	Scale

References belonging to Table 2 can be found in Appendix S4. Children (0–18 years), adults (>18–60 years), older adults (>60 years). MQ: Maastricht Questionnaire; CES-D: Center for Epidemiologic Studies–Depression Scale; SF-36: Short Form 36 Questionnaire; FSS: Fatigue Severity Scale; VAS-F: Visual Analogue Scale fatigue; GDS: Geriatric Depression Scale; EORTC: European Organisation for the Research and Treatment of Cancer quality of life questionnaire; BDI: Beck Depression Inventory; K10: Kessler Psychological Distress Scale; PHQ: Patient Health Questionnaire; 12 SF: 12-Item Short Form Health survey; MOB-T: Mobility Tiredness Scale; LIMB-T: Lower Limb Tiredness Scale; BORG: Borg Rating of Perceived Fatigue Scale; PFS: Pittsburgh Fatigability Scale; MFI-20: Multidimensional Fatigue Inventory; STI-CF: State–Trait Inventory for Cognitive Fatigue; CIS-T: Checklist individual strength; FACIT-F: Functional Assessment of Chronic Illness Therapy–Fatigue; 8SQ: Eight State Questionnaire; CHIPS: Cohen–Hoberman Inventory of Physical Symptoms; PIPER: Piper Fatigue Scale; CFQ: Chronic Fatigue Syndrome Questionnaire; GHQ: General Health Questionnaire; PROMIS: Patient-Reported Outcomes Measurement Information System; MFI-SF: Multidimensional Fatigue Symptom Inventory–Short Form; BFI: Brief Fatigue Inventory; FAS: Fatigue Assessment Scale; FIS: Fatigue Impact Scale; Chalder: Chalder Fatigue Questionnaire; MFS: Mental Fatigue Scale; DMFS: Dutch Multifactor Fatigue Scale; FSQ: Functional Status Questionnaire; SFS: Situational Fatigue Scale; FAI: Fatigue Assessment Instrument; BMFQ: Brief Mental Fatigue Questionnaire; FSI: Fatigue Symptom Inventory; DUFS: Dutch Fatigue Scale; DEFS: Dutch Exertion Fatigue Scale; CaFS: Cancer Fatigue Scale; SCFS: Schwartz Cancer Fatigue Scale; SOFA: Schedule of Fatigue and Anergia; CRFDS: Cancer Related Fatigue Distress Scale; FIS-D: Fatigue Impact Scale for Daily Administration; CHQ: Chronic Heart Failure Questionnaire; CRQ: Chronic Respiratory Questionnaire; ESAS: Edmonton Symptom Assessment System; FasD: Fatigue Associated with Depression Questionnaire; MAF: Multidimensional Assessment of Fatigue; NFI-MS: Neurological Fatigue Index for Multiple Sclerosis; PQ: Perform Questionnaire; POMS: Profile of Mood State–Fatigue; PROMIS: PROMIS-Fatigue; QOLIE: Quality of Life Inventory in Epilepsy; SCFS: Sleep Impact Scale; TOI-FCST: Trial Outcome Index-Fatigue; 30sCST: 30 second sit to stand test; UEE: Upper Extremity Exhaustion; GW: GripWork; FR: Fatigue Resistancetest.

**Table 3:** Instruments designed for measuring fatigue

Fatigue instrument	Construct Acute/chronic	Reference period	Assessment method	Targeted population and conditions	Aim	Reliability	Investigated validity	Responsiveness	Predictive validity on longevity	Completeness of underlying evidence
**Self-perceived fatigue**										
**BFI**	General Acute	Last 24 hours	Self-assessment	Older adults General condition, cancer	Evaluative	ICC: + [33] Cronbach α: + [26, 28, 33, 46] Test–retest: + [33]	Construct: + [33], − [26, 28] Cultural: NA Criterion: + [26, 33] Content: + [33] Discriminative:+ [46]	NA	NA	7/9
**FAS**	General Acute	Usually	Self-assessment	Older adults General population	Evaluative	ICC: + [46] Cronbach α: NA Test–retest: [46]	Construct + [46] Cultural: NA Criterion: NA Content: NA Discriminative: NA	MCID = 3.16–8.76 [72] MID: 3.0–4.2 [27]	NA	8/9
**FIS**	Physical, cognitive, psychosocial Acute	Present	Self-assessment	Older adults General population, cancer, multiple sclerosis	Evaluative	ICC: + [33, 46] Cronbach α: + [26, 33, 46] Test–retest: + [33, 46]	Construct: + [26, 33], − [46] Cultural: NA Criterion: + [33] Content: + [33] Discriminative: + [46]	MID: 4.8–17.3 [27]	NA	8/9
**FSS**	General Acute	Past week	Self-assessment	Older adults Not specified, multiple sclerosis, systemic lupus erythematosus	Evaluative	ICC: + [26] Cronbach α: + [33] Test–retest: + [26, 33]	Construct:— [26, 33] Cultural: NA Criterion: + [33] Content: + [33] Discriminative: + [26]	MID: 0.2–1.2 [27]	Falls [25]	9/9
**VAS-F**	General, energy Acute	Present	Self-assessment	Older adults General population, physical morbidities	Evaluative	ICC: + [26, 46] Cronbach α:+ [26, 28] Test–retest: NA	Construct: − [26] Cultural: NA Criterion: + [26, 46] Content: NA Discriminative: + [46]	MID: 1.4–13.9 [27]	Falls [25]	9/9
**MOB-T/LIMB-T**	Physical Acute	Present	Self-assessment	Older adults General population	Evaluative	Agreement: + [13] Kappa: + [13] ICC: + [13] Test–retest: NA	Construct: + [13, 28] Cultural: NA Criterion: + [13, 28] Content: − [13] Discriminative: NA	NA	Mortality [15] Hospitalisation [15] Falls [15] Decline ADL [15] Physical functioning [15] Heart disease [15]	8/9
**PFS**	Physical, mental Acute	Present	Self-assessment	Older adults General population	Evaluative	Cronbach α: + [26, 35, 73] ICC: + [73] Test–retest: + [26, 35, 73]	Construct + [28, 73] Cultural: NA Criterion: NA Content: NA Discriminative: + [73]	NA	Physical functioning [15]	9/9

*(Continued)*

**Table 3:** Continued

Fatigue instrument	Construct Acute/chronic	Reference period	Assessment method	Targeted population and conditions	Aim	Reliability	Investigated validity	Responsiveness	Predictive validity on longevity	Completeness of underlying evidence
**MFI-20**	General, physical, reduced activity, reduced motivation, mental Acute	Previous days	Self-assessment	Older adults General population, medical population	Evaluative	ICC: + [33, 46] Cronbach α: + [26, 33, 46] Test–retest: + [46]	Construct: + [22, 26, 33, 46] Cultural: NA Criterion: − [33] Content: − [33] Discriminative: + [26, 46]	MCID = 16.6 [74] MID: 11.5–13.3 [27] MID: 1.4–2.4 per subscale [27]	NA	8/9
**CIS**	General, concentration, reduced motivation, reduced activity Acute	2 weeks	Self-Assessment	Older adults General population, multiple sclerosis, Rheumatoid arthritis	Evaluative	ICC: + [28] Cronbach α: + [33, 75] Test–retest: + [28, 75]	Construct: − [28, 33] Cultural: NA Criterion: + [33, 75] Content: + [33] Discriminative: NA	NA	NA	7/9
**PIPER**	General Acute	Now	Self-assessment	Older adults General population, cancer	Evaluative	ICC: + [46] Cronbach α: + [26, 28, 33] Test–retest: + [33]	Construct: − [28, 33, 46] Cultural: NA Criterion: − [33] Content: + [33] Discriminative: NA	NA	NA	7/9
**FACIT-F**	General, physical Acute	Past week	Self-assessment	Older adults General population, cancer, stroke, HIV	Evaluative	Cronbach α: + [28, 33] ICC: + [46] Test–retest: + [28, 33]	Construct: + [33] [46] Cultural: NA Content: + [33] Criterion: + [28, 33] Discriminative: + [28]	MCID: 15.9 [74] MID: 3–8.3 [27]	NA	8/9
**CFQ**	Mental, physical Chronic	Past month	Self-assessment	Older adults General, physical and psychiatric morbidities	Evaluative/ discriminative	ICC: + [46] Cronbach α: + [33] Test–retest: + [26]	Construct: + [33] Cultural: NA Content: + [33] Criterion:? Discriminative: NA	MID: 2.3–3.3 [27]	NA	8/9
**FAI**	General, physical, mental Acute	Past 2 weeks	Self-assessment	Older adults General, physical and psychiatric morbidities	Evaluative	ICC: NA Cronbach α: + [26] Test–retest: + [26]	Construct: + [26] Cultural: NA Criterion: NA Content: NA Discriminative: NA	NA	NA	7/9
**FSI**	General, physical, mental Acute	Past week	Self-assessment	Older adults General, cancer	Evaluative	ICC: + [46] Cronbach α: + [26] Test–retest: + [46]	Construct: + [26] [46] Cultural: NA Criterion: + [26] Content: NA Discriminative: + [26]	NA	NA	7/9
**DUFS**	Physical Acute	Now	Self-assessment	Older adults General	Discriminative	ICC: + [26] Cronbach α: NA Test–retest: NA	Construct: + [26] Cultural: NA Criterion: + [26] Content: NA Discriminative: NA	NA	NA	7/9

*(Continued)*

**Table 3:** Continued

Fatigue instrument	Construct Acute/chronic	Reference period	Assessment method	Targeted population and conditions	Aim	Reliability	Investigated validity	Responsiveness	Predictive validity on longevity	Completeness of underlying evidence
**DEFS**	Physical Acute	Now	Self-assessment	Older adults General	Discriminative	ICC: NA Cronbach α: + [26] Test–retest: NA	Construct: − [26] Cultural: NA Criterion: − [26] Content: NA Discriminative: NA	NA	NA	7/9
**FIS-D**	General Acute	Now	Self-assessment	Older adults General, multiple sclerosis	Evaluative	ICC: + [33] Cronbach α: + [26, 33] Test–retest: + [33]	Construct: − [26, 33] Cultural: NA Criterion: + [33], − [26] Content: + [33] Discriminative: NA	MID: 4.8–17.3 [27]	NA	8/9
**MAF**	General, physical, mental Acute	Past week	Self-assessment	Older adults General Rheumatoid arthritis	Evaluative	ICC: NA Cronbach α: + [33] Test–retest: + [33]	Construct: + [33] Cultural: NA Criterion: + [33] Content: + [33] Discriminative: NA	MID: 5.0–9.2 [27]	NA	8/9
**PROMIS-F**	General Acute	Past week	Self-assessment	Older adults General	Evaluative	ICC: + [76] Cronbach α: + [76] Test–retest: NA	Construct: + [76] Cultural: NA Criterion: NA Content: NA Discriminative: NA	MID (ES): 0.34–0.79 [27]	NA	8/9
**Muscle fatigue**										
**12-minute walk test**	Physical Acute	Now	Objective assessment	Older adults General, respiratory diseases, cardiovascular disease, Parkinson, COPD	Evaluative	ICC: + [77] Cronbach α: NR Test–retest: + [77]	Construct: NA Cultural: NA Criterion: + [77] Content: NA Discriminative: NA	NA	NA	7/9
**FR**	Physical Acute	Now	Objective assessment	Older adults General, healthy older adults	Evaluative	ICC: + [45] Cronbach α: NR Test–retest: NA	Construct: NA Cultural: NA Criterion: NA Content: NA Discriminative: NA	NA	NA	7/9
**GW**	Physical Acute	Now	Objective assessment	Older adults General, healthy older adults	Evaluative	ICC: +/− [63] Cronbach α: NR Test–retest: + [63]	Construct: − [47, 78] Cultural: NA Criterion: + [47, 78] Content: NA Discriminative: NA	SDC: 1,376.36 Kpa*s [63]	NA	8/9

BFI: Brief Fatigue Inventory; FAS: Fatigue Assessment Scale; FIS: Fatigue Impact Scale; FSS: Fatigue Severity Scale; VAS-F: Visual Analogue Scale fatigue; MOB-T: Mobility Tiredness scale; LIMB-T: Lower Limb Tiredness scale; PFS: Pittsburgh Fatigability Scale; MFI-20: Multidimensional Fatigue Inventory; CIS: Checklist individual strength; PIPER: Piper Fatigue Scale; FACIT-F: Functional Assessment of Chronic Illness Therapy–Fatigue; CFQ: Chalder Fatigue Questionnaire; FAI: Fatigue Assessment Instrument; FSI: Fatigue Symptom Inventory; DUFS: Dutch Fatigue Scale; DEFS: Dutch Exertion Fatigue Scale; FIS-D: Fatigue Impact Scale for Daily Administration; MAF: Multidimensional Assessment of Fatigue; PROMIS-F: PROMIS-Fatigue; GW: GripWork; FR: Fatigue resistance test; ADL: activities of daily living; kPa: kilopascal; MID: minimal important difference; MCID: minimal clinical important difference; SDC: smallest detectable change. Construct (convergent or divergent) validity is the degree to which scores of a measurement instrument are consistent with the hypothesis [20]. Content validity assesses the degree to which the content of a measurement instrument is an adequate reflection of the construct to be measured [20]. Criterion validity demonstrates the correlation between the measurement of interest and a gold standard. Concurrent validity shows you the extent of the agreement between two measures or assessments taken at the same time. Validity was indicated as +: *r* > 0.70, −: *r* < 0.70,?: not all information for ‘+’ reported, NA: not assessed; NR: not relevant. Reliability was indicated as +: ICC or weighted Kappa >0.70, −: ICC or weighted Kappa <0.70,?: ICC or weighted Kappa not reported, NA: not assessed.

### Measurement properties and quality of the evidence

No studies reported data on cross-cultural or structural validity.

### Content validity

For 11 instruments, content validity was reported. Content validity was low for the MOB-T [13] and the MFI-20 [33]. There was sufficient high-quality evidence for the comprehensiveness of the BFI [33], FIS [33], FSS [33], CIS [33], PIPER [33], FACIT-F [33], CFQ [33], FIS-D [33] and the MAF [33] (Table 3). For the remaining instruments, content validity was not reported.

### Construct validity

Construct validity was reported for 19 fatigue instruments [34–45], and good construct validity was reported for 11 instruments [34–42] supported by high pooled coefficients for the correlations (*r* > 0.70). Mota and Pimenta [26] reported good construct validity for the BFI and they found a strong correlation between the BFI and FACIT-F (*r* = −0.9) while others reported a moderate correlation between the BFI and anaemia (*r* = 0.36) [33]. The FIS showed good construct validity with the SF-36 (*r* = 0.8) [46] and low pooled coefficients for the correlations for general health (*r* = 0.53) [26, 33]. Low pooled coefficients for the correlations were found for the FSS [26], VAS-F [26], CIS [28, 33], DEFS [26], FIS-D [26, 33] and GW [47]. Based on these findings, the overall construct validity of the fatigue instruments was rated as moderate (Table 3).

### Criterion validity

Criterion validity was reported for 17 instruments [31, 32, 34, 36–39, 41, 42, 48–52], of which 12 instruments [31, 32, 34, 37–39, 41, 42, 48–51] reported high-quality evidence of sufficient criterion validity and 2 (MFI-20 [36] and FIS-D [52]) reported low criterion validity (see Table 3). The CFQ did not show all information for reporting ‘+’ and for the FIS-D high criterion validity was reported (*r* = 0.8) by Machado *et al*. [33] where criterion validity was tested through its correlation with FSS (*r* = 0.75), and Mota and Pimenta [26] showed moderate criterion validity between the FIS-D and a numeric scale (*r* = 0.5).

### Internal consistency and reliability

Internal consistency was good for 19 fatigue instruments [31, 32, 34–39, 41–45, 47–50, 52, 53] (ICC > 0.7). For three instruments (FAI, DEFS and MAF), the ICC was not reported. The subscale values of Cronbach’s alpha were high (Cronbach α > 0.7) for 17 instruments [31, 32, 34–42, 48–53] and was not reported for two instruments (FAS and DUFS). Construct validity was considered not relevant for the 12-minute walk test, FR and GW since these are 1-item physical tests. Test–retest validity was high (>0.7) for 15 instruments [31, 35–41, 43, 44, 48–52] and was not reported for 7 instruments [32, 34, 42, 45, 47, 53].

### Responsiveness

For only 10 instruments [35, 36, 38, 39, 43, 49, 51–53] responsiveness (Minimal clinical important difference) was reported.

### Predictive validity

For two fatigue instruments (MOB-T and PFS), predictive value of fatigue on different health outcomes was found. Older adults who feel fatigued, as measured with the MOB-T [34], have an increased risk for mortality (OR 2.29 [1.67–3.14]), hospitalisation (OR 2.20 [1.10–4.40]), falls (OR 2.40 [1.30–4.40]), heart disease and decline in ADL (OR 2.30 [1.30–4.10]), and physical functioning (OR 6.50 [2.70–15.60]). For the PFS [35], significant odds ratios were found for an increased risk for decreased physical functioning (OR 2.54 [1.58–4.10]) (Table 3).

**Table 4:** Fatigue instruments characteristics in relation toVC

	1	2	3	4	5	6	7	8
Fatigue instrument	Feasible to quantify biomarkers or proxy markers	Feasible to measure or collect in low-resource settings	Useful and informative for monitoring	Distinct attribute	Acceptable cost and resource demand	Sufficient availability and no ethical concerns	Implementable	Robust psychometric properties
BFI	Yes	Yes	Unclear	Yes	Yes	Yes	Yes	Yes
FAS	Yes	Yes	Yes	Yes	Yes	Yes	Yes	Yes
FIS	Yes	Yes	Unclear	Yes	Yes	Yes	Yes	Yes
FSS	Yes	Yes	Yes	Yes	Yes	Yes	Yes	Yes
VAS-F	Yes	Yes	Yes	Yes	Yes	Yes	Yes	Yes
MOB-T/LIMB-T	Yes	Yes	Yes	Yes	Yes	Yes	Yes	Yes
PFS	Yes	Yes	Yes	Yes	Yes	Yes	Yes	Yes
MFI-20	Yes	Yes	Yes	Yes	Yes	Yes	Yes	Yes
CIS	Yes	Yes	Unclear	Yes	Yes	Yes	Yes	Yes
PIPER	Yes	Yes	Unclear	Yes	Yes	Yes	Yes	Yes
FACIT-F	Yes	Yes	Yes	Yes	No	Yes	Yes	Yes
CFQ	Yes	Yes	Yes	Yes	Yes	Yes	Yes	Yes
FAI	Yes	Yes	Unclear	Yes	Yes	Yes	Yes	Unclear
FSI	Yes	Yes	Unclear	Yes	Yes	Yes	Yes	Yes
DUFS	Yes	Yes	Unclear	Yes	No	Yes	Yes	Unclear
DEFS	Yes	Yes	Unclear	Yes	No	Yes	Yes	Unclear
FIS-D	Yes	Yes	Yes	Yes	Yes	Yes	Yes	Yes
MAF	Yes	Yes	Yes	Yes	Yes	Yes	Yes	Yes
PROMIS-F	Yes	Yes	Yes	Yes	Yes	Yes	Yes	Yes
12-minute walk test	Yes	Yes	Yes	Yes	Yes	Yes	Yes	Yes
Fatigue resistance test	Yes	No	Unclear	Yes	No	Yes	Unclear	Unclear
GripWork	Yes	No	Unclear	Yes	No	Yes	Unclear	Unclear

The scales were graded as yes, unclear,no.

^a^Feasible to quantify biomarkers or proxy markers: not too many items, easy to fillin.

^b^Feasible to measure or collect in low-resource settings.

^c^Useful and informative for monitoring; responsive to change.

^d^Distinct attribute: discriminates cases from non-cases with acceptable level of sensitivity and specificity.

^e^Acceptable cost and resource demand.

^f^Sufficient availability and no ethical concerns.

^g^Implementable, usability: easy to understand, easy to complete.

^h^Robust psychometric properties: reliable, reproducibility, accurate.

### Fatigue instruments for implementation in VC

Table 4 shows the characteristics of the fatigue instruments in context of criteria for VC. Twelve instruments [32, 34–36, 38, 39, 43, 44, 51–54] are useful and informative for monitoring, while for the others this was unclear. All instruments are feasible to quantify, are a distinct attribute and show robust psychometric properties. The FAS, FSS, VAS-F, MOB-T, MFI, CFQ, FIS-D, MAF, PROMIS and the 12-minute walking test scored good on all criteria. The FR and GW both use an instrument, which makes them less implementable in community settings. The FSS, VAS-F, FACIT-F, FIS and the PFS could be used in the context of VC. The FIS includes 40 items and is designed to measure cognitive and physical fatigue but is rather long. The FACIT-F is a licensed scale that complicates the usability in low-resource settings; on the contrary, the scale is available in 76 different languages. The FSS is a unidimensional instrument while the PFS, FIS and VAS-F comprised two dimensions of fatigue: the VAS-F included fatigue and energy while the PFS looks at physical and mental fatigue.

## Discussion

This umbrella review is a comprehensive summary of the literature on fatigue instruments and aimed to identify available scales suitable for measuring fatigue in community-dwelling older adults and to review their measurement properties. The results indicate that there are several fatigue instruments that can be used for measuring the E&M domain in the context of VC, and that these instruments are useful and informative for monitoring. The FSS, VAS-F, FACIT-F, FIS and the PFS scored relatively well on almost all items and in addition demonstrated associations with negative health outcomes. Therefore, these instruments could be used as biomarkers of E&M in the context ofVC.

Evidence on psychometric properties was limited and evidence for community-dwelling older adults was scarce. To measure change in trajectories of fatigue, good test–retest reliability and responsiveness are vital, thus future studies should focus on test–retest reliability of fatigue instruments in community-dwelling older adults. For the FAS, FIS, FSS, VAS-F, MFI-20, FACIT-F, CFQ, FIS-D, MAF and the PROMIS-F, minimum important differences were reported in the literature [27]. However, assessing responsiveness can be challenging since effective interventions to counter fatigue in older adults are scarce [55]. Non-pharmacological interventions (i.e. mindfulness, muscle relaxation, yoga, Tai Chi, behavioural therapy) have shown some positive effects [56], but further research is needed to develop better interventions to influence fatigue in older adults. Other instruments are also promising for use in the context of E&M, and the MOB-T, for example, met 8/9 criteria; however, the MOB-T is more often used in populations with mobility problems [57], which makes it less suitable for measuring trajectories in VC. The MFI-20 [36] is also a very promising scale and is a widely adopted instrument in various populations [46, 58, 59] and comprises different fatigue constructs. This scale shows good reliability and validity, but there is insufficient information regarding responsiveness of this scale. In total, 10 scales (Table 2) were not validated for older adults, and more research in older populations is required regarding these scales since they may be promising for measuring fatigue in older adults.

The lack of a gold standard to measure fatigue [28] leads to a great heterogeneity of fatigue instruments in the literature. The complex and ubiquitous nature of fatigue challenges evaluating fatigue in a standardised manner and makes it complicated to compare fatigue instruments. Self-perceived fatigue instruments included either one domain of fatigue or multiple domains of fatigue. Choice of a fatigue instrument should be carefully considered depending on the aim [13]. Most of the identified instruments included self-perceived fatigue, measuring different domains, while the muscle fatigability instruments were objective and focus on one fatigue domain. More self-perceived fatigue instruments were identified compared to muscle fatigability, in line with earlier research [15, 16], where also only few muscle fatigue instruments were identified. However, evidence showed that muscle fatigue is of high importance for activities of daily living and frailty [60]. Van Geel *et al*. [24] identified seven different walking tests for measuring walking-related performance fatigability. However, walking performance can be limited by other factors than fatigue alone, such as cardiovascular function [61], and could be therefore seen more as a test for measuring endurance rather than fatigue. As such, these tests are more closely related to locomotor capacity [62] and should be considered performance tests rather than tests for fatigue. In contrast, GW and FR measure neuromuscular activation and are more suitable for measuring and monitoring muscle fatigability [14, 63]. However, more research on the predictive validity of these instruments is required.

Many instruments measured fatigue-related constructs such as depression, quality of life and pain. Depressive symptoms were assessed in eight fatigue instruments (CES-D, GDS, BDI, K10, PHQ, 8SQ, GHQ, PROMIS), quality of life in three instruments (SF-36, SF-12, EORTCQLQ-C3) and physical activity in three (INTERAI, Borg, CFQ). Different interchangeable symptoms are related to fatigue with conflicting overlapping concepts [13]. Research showed that there is a large overlap between self-perceived fatigue and mental or psychological state [64], especially between fatigue and depression [12]. Other mechanisms could also be related to fatigue; for instance, some aspects of fatigue might result from poor sleep [13, 65]. Fatigue is an indicator of frailty and is closely related to frailty since it is one of the key domains in the concept of physical frailty [66]. Half of frailty scales include at least one fatigue item; however, fatigue seems to be assessed mostly through a single item derived from fatigue instruments [16]. In contrast, within the context of VC, fatigue will be assessed through a full fatigue instrument, highlighting the difference in assessing fatigue in the context of frailty andVC.

While most fatigue instruments have been validated in populations with chronic conditions, such as cancer survivors, patients with multiple sclerosis and those with rheumatoid arthritis, the relationship between fatigue and negative health outcomes has also been established [15]. The presence of fatigue increases the risk of physical decline, hospitalisation, diseases and mortality in community-dwelling older adults [15]. Therefore, fatigue may be a proxy for an underlying process causing adverse health outcomes [67]. Fatigue includes characteristics that may be explained by central mechanisms and may be different in various medical conditions. The functions carried out in the context of energy provision by the mitochondrion are subject to the ageing process [4] and alterations in mitochondrial function may explain the severity of fatigue symptoms [5], although strong direct evidence is lacking to date. Nevertheless, mitochondrial dysfunction is one of the hallmarks of ageing and can result in metabolic and degenerative diseases [68]. The Ca^2+^ release channels are one of the targets for oxidative damage, thus influencing Ca^2+^ release and muscle force generation capacity, which has effects on longevity [68]. Degeneration of mitochondria is linked to senescence and inflammation [68, 69] which are also pathophysiological factors associated with fatigue. Research shows that shorter telomere length, indicating advanced ageing, is associated with greater fatigability in older adults [7]. Therefore, fatigue is associated with a variety of biological changes and outcomes related to ageing.

Designing a fatigue index that combines both self-perceived fatigue and muscle fatigability could provide a more comprehensive assessment of fatigue in the context of VC. This approach would consider both subjective and objective aspects of fatigue, which could improve the accuracy and clinical relevance of the assessment. Research has shown that individuals showing high muscle fatigability (i.e. poor muscle endurance) and high feelings of self-perceived fatigue are a clinical subgroup that are more prone to develop frailty [70]. Other studies showed that when high muscle fatigability is associated with high self-perceived fatigue, this predicts decline in activities of daily living and gait speed [71]. The combination of self-perceived fatigue and muscle fatigability in a fatigue index has the potential to provide a more comprehensive and clinically relevant assessment of fatigue in the context ofVC.

Overall, the quality of the articles included in this umbrella review was moderate to good. None of the reviews provided a list of included and excluded studies and only five studies reported on the methodological quality of the original studies. This could be a limitation for the umbrella review since the evidence provided was not methodologically checked in all the included reviews. Since we included only systematic reviews and meta-analysis, we might have missed fatigue instruments reported in original studies only; however, seven of the included articles were published in the past 5 years, suggesting that the most recent fatigue scales have been identified. This review focused on community-dwelling older adults excluding fatigue in specific medical diseases. It can be difficult to ascribe fatigue to a single disease or cause, and for this reason, we excluded articles with hospitalised patients or patients with specific health conditions. Conceptually, it can be assumed that hospitalised and/or institutionalised older adults are possibly at an already more advanced stage of fatigue. For this reason, the psychometric properties of the instruments may differ in a general population as opposed to patients with chronic diseases, as most data were available only in the latter group. Despite these limitations, the review provides a comprehensive overview of 21 available fatigue instruments that can be used in community-dwelling older adults and serves as a guideline for healthcare professionals and policy makers in identifying older people atrisk.

WHO indicated that developing standardised, valid and objective tools to assess and monitor VC needed to be addressed before VC can be an integral part of healthcare systems designed to promote HA [3]. This is a first review validating measurements in the E&M domain for evaluating VC. More research to identify other measurement tools that can be used for the E&M domain and the other domains of VC will be done. When suitable measures for all the domains of VC (energy and metabolism, neuromuscular function and immune and stress response) are available on population level, targeted interventions for boosting VC and to prevent age-related diseases can be developed.

## Conclusion

This umbrella review summarises the available fatigue instruments for older adults that can be used in a community setting. The fatigue instruments are analysed based on their psychometric properties, including validity and reliability. The study found that the Fatigue Severity Scale (FSS), Pittsburgh Fatigability Scale (PFS), Functional Assessment of Chronic Illness Therapy Fatigue (FACIT-F), Visual Analogue scale (VAS-F) and Fatigue Impact Scale (FIS) are the most effective instruments for measuring fatigue in older adults and can be used in VC. The study emphasises the importance of measuring VC in adults since fatigue is linked to biological changes of ageing and highlights the need for a more preventive approach in healthcare. This is the first review to identify fatigue tools for measuring E&M in the context of VC; more work on other biomarkers for VC is necessary to accurately measure and evaluate VC in populations.

## Supplementary Material

aa-23-0367-File002_afad140

## Data Availability

The authors confirm that the data supporting the findings of this study are available within the article and/or its supplementary materials.
