# Supplementary material for: Measurement properties of instruments to measure the fatigue domain of vitality capacity in community-dwelling older people: an umbrella review of systematic reviews and meta-analysis
Source: Age Ageing. 2023 Oct 30;52(Suppl 4):iv26–43. doi: 10.1093/ageing/afad140 (PMC10615047; doi:10.1093/ageing/afad140)
Supplement: aa-23-0367-File002_afad140 [file aa-23-0367-file002_afad140.docx]

World Health Organisation *Measurements of Healthy Ageing.*

Measurement properties of instruments to measure the fatigue domain of vitality capacity community-dwelling older people: An umbrella review of systematic reviews and meta-analysis.

SUPPLEMENTARY DATA

- **Appendix 1.** Inclusion & Exclusion of articles
- **Appendix 2.** Methodology of data extraction
- **Appendix 3.** Risk of bias summary: review authors' judgements about each risk of bias item for each included study.
- **Appendix 4:** References belonging to table 2.

**Appendix 1. Inclusion & Exclusion of articles**

**
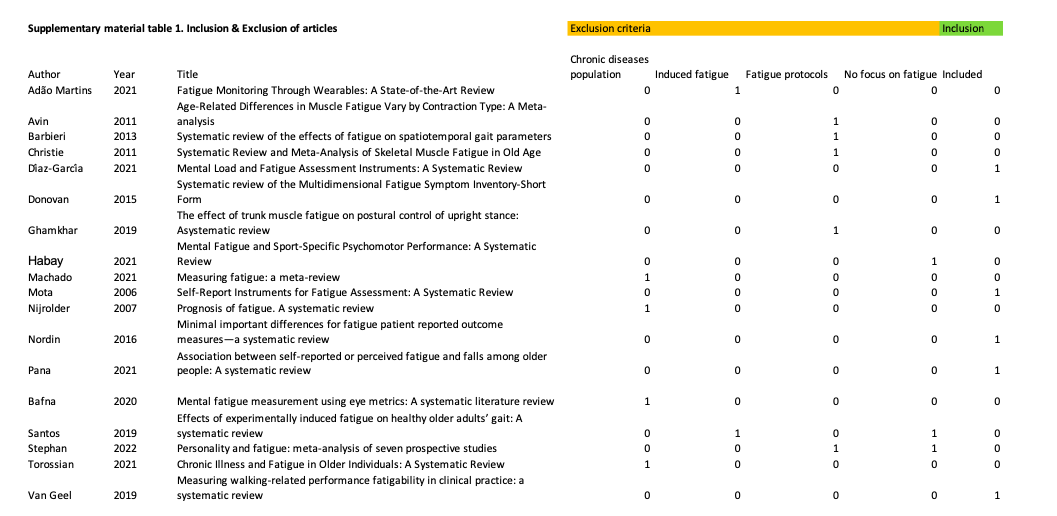
**

**Note: 0: negative, 1: positive**

**Appendix 2. Methodology of data extraction**


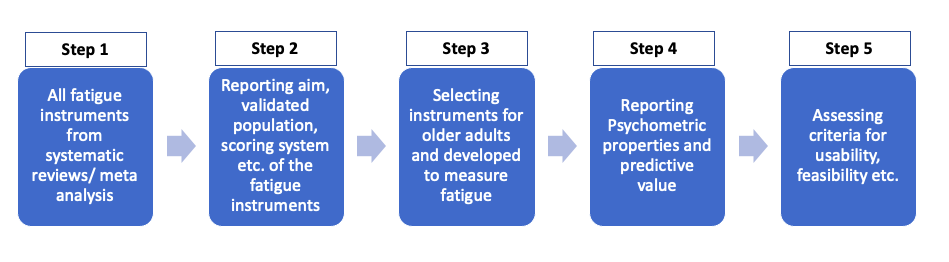


Note:

**Step 1**. Selection of all fatigue instruments reported in the systematic reviews and meta-analyses were extracted and divided into self-perceived fatigue and muscle fatigue instruments. The following data were extracted: study, population (i.e., age, gender), countries included, the fatigue instruments, reported psychometric properties (yes/no) and predictive validity (yes/no) (table 1).

**Step 2:** Reporting data on the aim of the fatigue instruments, validated populations (children, adults and older adults), scoring system, number of items and whether the fatigue instruments were designed for fatigue (yes/no) (table 2).

**Step 3:** Selection of fatigue instruments primarily designed for fatigue and validated in older adults. To verify whether the instruments was designed for fatigue, we used the following definition: “Fatigue is a state usually associated with a weakening or depletion of one's physical and/or mental resources, ranging from a general state of lethargy to a specific, work-induced burning sensation within one's muscles”. It leads to the inability to continue functioning at a normal level of activity”(13). The selected fatigue instruments are shown in table 3.

**Step 4:** The selected instruments (designed for fatigue and validated in older adults) were assessed for fatigue construct, reference period, assessment method, target population, aim, reliability, validity, responsiveness, and predictive validity on longevity (table 3)

**Step 5:** The fatigue instruments were assessed (dichotomously (yes/no and unclear) by the following criteria 1) feasibility to quantify biomarkers or proxy biomarkers, 2) feasibility to measure or collect in low-resource settings, 3) usefulness and informativeness for monitoring, 4) distinctiveness instrument, 5) acceptability regarding cost and resource demand, 6) availability and no ethical concerns and 7) implementability, 8) robustness regarding psychometric properties (table 4).

**Appendix 3. Risk of bias summary: review authors' judgements about each risk of bias item for each included study.**

**
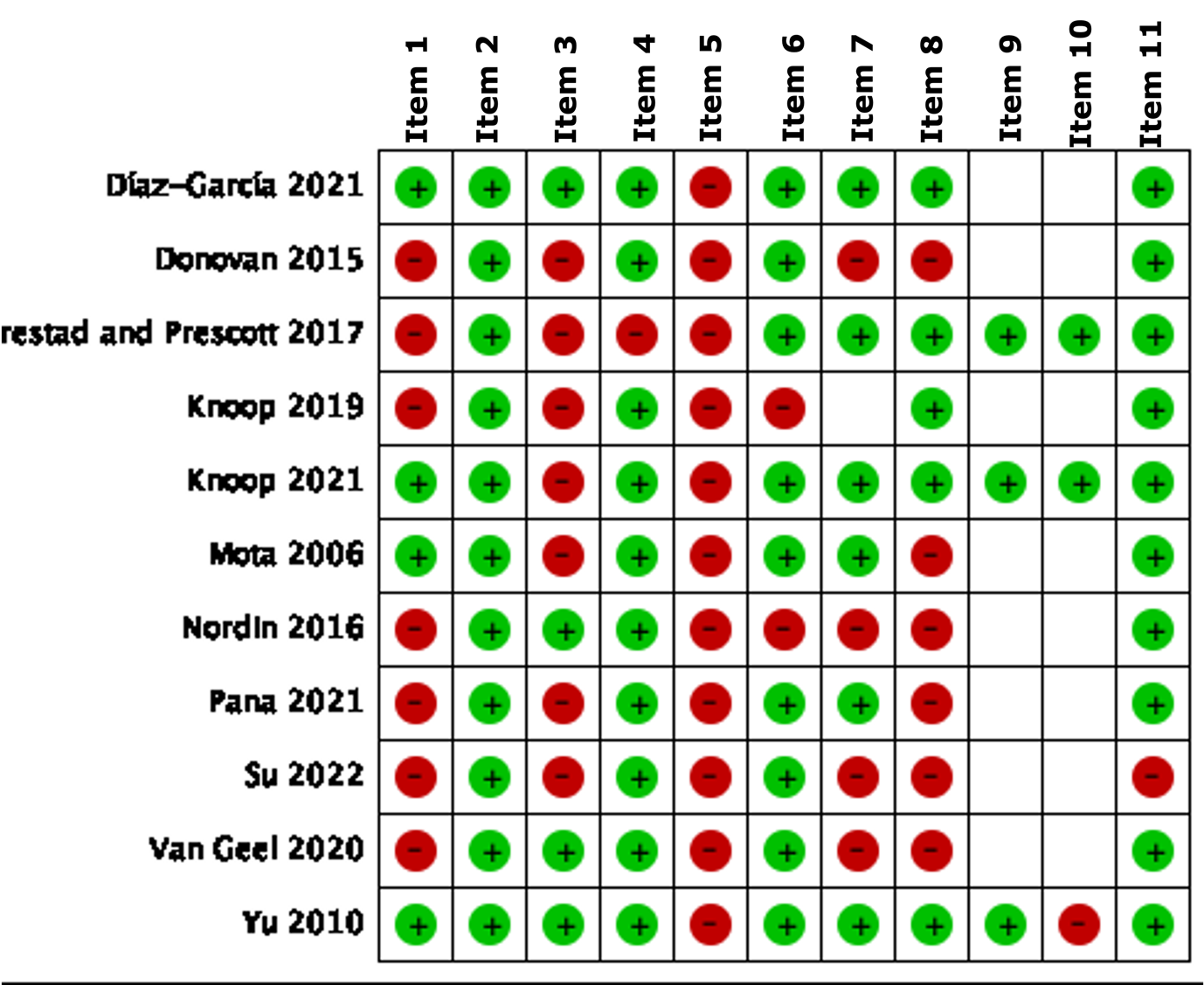
**

1. Was an 'a priori' design provided
2. Was there duplicate study selection and data extraction?
3. Was a comprehensive literature search performed?
4. Was the status of publication (i.e. grey literature) used as an inclusion criterion?
5. Was a list of studies (included and excluded) provided?
6. Were the characteristics of the included studies provided?
7. Was the scientific quality of the included studies assessed and documented?
8. Was the scientific quality of the included studies used appropriately in formulating conclusions?
9. Were the methods used to combine the findings of studies appropriate?
10. Was the likelihood of publication bias assessed?
11. Was the conflict of interest included?

**Appendix 4: References belonging to table 2.**

Aaronson, N. K., Ahmedzai, S., Bergman, B., Bullinger, M., Cull, A., Duez, N. J., Filiberti, A., Flechtner, H., Fleishman, S. B., de Haes, J. C., & et al. (1993). The European Organization for Research and Treatment of Cancer QLQ-C30: a quality-of-life instrument for use in international clinical trials in oncology. *J Natl Cancer Inst*, *85*(5), 365-376. https://doi.org/10.1093/jnci/85.5.365

Appels, A., Höppener, P., & Mulder, P. (1987). A questionnaire to assess premonitory symptoms of myocardial infarction. *Int J Cardiol*, *17*(1), 15-24. https://doi.org/10.1016/0167-5273(87)90029-5

Arai, M. (2021). Evaluating the usefulness of Ninjin'yoeito Kampo medicine in combination with rehabilitation therapy in patients with frailty complicated by intractable dizziness. *Neuropeptides*, *90*, 102189. https://doi.org/10.1016/j.npep.2021.102189

Avlund, K., Kreiner, S., & Schultz-Larsen, K. (1993). Construct validation and the Rasch model: functional ability of healthy elderly people. *Scand J Soc Med*, *21*(4), 233-246. https://www.ncbi.nlm.nih.gov/pubmed/8310276

Baró, E., Carulla, J., Cassinello, J., Colomer, R., Mata, J. G., Gascón, P., Gasquet, J. A., Herdman, M., Rodríguez, C. A., Sánchez, J., & Valentín, V. (2009). Development of a new questionnaire to assess patient perceptions of cancer-related fatigue: item generation and item reduction. *Value Health*, *12*(1), 130-138. https://doi.org/10.1111/j.1524-4733.2008.00426.x

Bautmans, I., & Mets, T. (2005). A fatigue resistance test for elderly persons based on grip strength: Reliability and comparison with healthy young subjects. *Aging Clinical and Experimental Research*, *17*(3), 217-222. https://doi.org/10.1007/BF03324600

Beck, A. T., Ward, C. H., Mendelson, M., Mock, J., & Erbaugh, J. (1961). An inventory for measuring depression. *Arch Gen Psychiatry*, *4*, 561-571. https://doi.org/10.1001/archpsyc.1961.01710120031004

Belza, B. L. (1995). Comparison of self-reported fatigue in rheumatoid arthritis and controls. *J Rheumatol*, *22*(4), 639-643.

Bentall, R. P., Wood, G. C., Marrinan, T., Deans, C., & Edwards, R. H. (1993). A brief mental fatigue questionnaire. *Br J Clin Psychol*, *32*(3), 375-379. https://doi.org/10.1111/j.2044-8260.1993.tb01070.x

Borg, G. (1990). Psychophysical scaling with applications in physical work and the perception of exertion. *Scand J Work Environ Health*, *16 Suppl 1*, 55-58. https://doi.org/10.5271/sjweh.1815

Boyle, G. (1991). Item Analysis of the Subscales in the Eight State Questionnaire (8SQ): Exploratory and Confirmatory Factor Analyses. *Multivariate Experimental Clinical Research*, *10*, 37-65.

Chalder, T., Berelowitz, G., Pawlikowska, T., Watts, L., Wessely, S., Wright, D., & Wallace, E. P. (1993). Development of a fatigue scale. *J Psychosom Res*, *37*(2), 147-153. https://doi.org/10.1016/0022-3999(93)90081-p

Chang, V. T., Hwang, S. S., & Feuerman, M. (2000). Validation of the Edmonton Symptom Assessment Scale. *Cancer*, *88*(9), 2164-2171. https://doi.org/10.1002/(sici)1097-0142(20000501)88:9<2164::aid-cncr24>3.0.co;2-5

Cohen, S., & Hoberman, H. M. (1983). Positive Events and Social Supports as Buffers of Life Change Stress1. *Journal of Applied Social Psychology*, *13*(2), 99-125. https://doi.org/https://doi.org/10.1111/j.1559-1816.1983.tb02325.x

Cramer, J. A., Perrine, K., Devinsky, O., Bryant-Comstock, L., Meador, K., & Hermann, B. (1998). Development and cross-cultural translations of a 31-item quality of life in epilepsy inventory. *Epilepsia*, *39*(1), 81-88. https://doi.org/10.1111/j.1528-1157.1998.tb01278.x

Crawford, B. B., S.; Burrell, A.; . (2007). Development and validation of the Sleep Impact Scale for insomnia. *Value Health*, *10*.

Csuka, M., & McCarty, D. J. (1985). Simple method for measurement of lower extremity muscle strength. *The American journal of medicine*, *78*(1), 77-81. http://www.ncbi.nlm.nih.gov/pubmed/3966492

De Dobbeleer, L., Beyer, I., Hansen, A. M., Molbo, D., Mortensen, E. L., Lund, R., & Bautmans, I. (2019). Grip Work Measurement with the Jamar Dynamometer: Validation of a Simple Equation for Clinical Use. *J Nutr Health Aging*, *23*(2), 221-224. https://doi.org/10.1007/s12603-019-1155-4

Engelhard, M. M., Dandu, S. R., Patek, S. D., Lach, J. C., & Goldman, M. D. (2016). Quantifying six-minute walk induced gait deterioration with inertial sensors in multiple sclerosis subjects. *Gait Posture*, *49*, 340-345. https://doi.org/10.1016/j.gaitpost.2016.07.184

Fisk, J. D., & Doble, S. E. (2002). Construction and validation of a fatigue impact scale for daily administration (D-FIS). *Qual Life Res*, *11*(3), 263-272. https://doi.org/10.1023/a:1015295106602

Fisk, J. D., Ritvo, P. G., Ross, L., Haase, D. A., Marrie, T. J., & Schlech, W. F. (1994). Measuring the functional impact of fatigue: initial validation of the fatigue impact scale. *Clin Infect Dis*, *18 Suppl 1*, S79-83. https://doi.org/10.1093/clinids/18.supplement_1.s79

Garcia, S. F., Cella, D., Clauser, S. B., Flynn, K. E., Lad, T., Lai, J. S., Reeve, B. B., Smith, A. W., Stone, A. A., & Weinfurt, K. (2007). Standardizing patient-reported outcomes assessment in cancer clinical trials: a patient-reported outcomes measurement information system initiative. *J Clin Oncol*, *25*(32), 5106-5112. https://doi.org/10.1200/jco.2007.12.2341

Gershon, R. C., Rothrock, N., Hanrahan, R., Bass, M., & Cella, D. (2010). The use of PROMIS and assessment center to deliver patient-reported outcome measures in clinical research. *J Appl Meas*, *11*(3), 304-314.

Glynn, N. W., Santanasto, A. J., Simonsick, E. M., Boudreau, R. M., Beach, S. R., Schulz, R., & Newman, A. B. (2015). The Pittsburgh Fatigability scale for older adults: development and validation. *J Am Geriatr Soc*, *63*(1), 130-135. https://doi.org/10.1111/jgs.13191

Goldberg, D. P., & Hillier, V. F. (1979). A scaled version of the General Health Questionnaire. *Psychol Med*, *9*(1), 139-145. https://doi.org/10.1017/s0033291700021644

Goldman, M. D., Marrie, R. A., & Cohen, J. A. (2008). Evaluation of the six-minute walk in multiple sclerosis subjects and healthy controls. *Mult Scler*, *14*(3), 383-390. https://doi.org/10.1177/1352458507082607

Guyatt, G. H., Nogradi, S., Halcrow, S., Singer, J., Sullivan, M. J., & Fallen, E. L. (1989). Development and testing of a new measure of health status for clinical trials in heart failure. *J Gen Intern Med*, *4*(2), 101-107. https://doi.org/10.1007/bf02602348

Hadzi-Pavlovic, D., Hickie, I. B., Wilson, A. J., Davenport, T. A., Lloyd, A. R., & Wakefield, D. (2000). Screening for prolonged fatigue syndromes: validation of the SOFA scale. *Soc Psychiatry Psychiatr Epidemiol*, *35*(10), 471-479. https://doi.org/10.1007/s001270050266

Hann, D. M., Jacobsen, P. B., Azzarello, L. M., Martin, S. C., Curran, S. L., Fields, K. K., Greenberg, H., & Lyman, G. (1998). Measurement of fatigue in cancer patients: development and validation of the Fatigue Symptom Inventory. *Qual Life Res*, *7*(4), 301-310. https://doi.org/10.1023/a:1024929829627

Hogeveen, S. E., Chen, J., & Hirdes, J. P. (2017). Evaluation of data quality of interRAI assessments in home and community care. *BMC Med Inform Decis Mak*, *17*(1), 150. https://doi.org/10.1186/s12911-017-0547-9

Holley, S. K. (2000). Evaluating patient distress from cancer-related fatigue: an instrument development study. *Oncol Nurs Forum*, *27*(9), 1425-1431.

Jette, A. M., Davies, A. R., Cleary, P. D., Calkins, D. R., Rubenstein, L. V., Fink, A., Kosecoff, J., Young, R. T., Brook, R. H., & Delbanco, T. L. (1986). The Functional Status Questionnaire: reliability and validity when used in primary care. *J Gen Intern Med*, *1*(3), 143-149. https://doi.org/10.1007/bf02602324

Johansson, B., & Rönnbäck, L. (2014). Evaluation of the Mental Fatigue Scale and its relation to Cognitive and Emotional Functioning after Traumatic Brain Injury or Stroke. *International Journal of Physical Medicine & Rehabilitation*, *2*. https://doi.org/10.4172/2329-9096.1000182

Kessler, R. C., Barker, P. R., Colpe, L. J., Epstein, J. F., Gfroerer, J. C., Hiripi, E., Howes, M. J., Normand, S. L., Manderscheid, R. W., Walters, E. E., & Zaslavsky, A. M. (2003). Screening for serious mental illness in the general population. *Arch Gen Psychiatry*, *60*(2), 184-189. https://doi.org/10.1001/archpsyc.60.2.184

Kroenke, K., Spitzer, R. L., & Williams, J. B. (2001). The PHQ-9: validity of a brief depression severity measure. *J Gen Intern Med*, *16*(9), 606-613. https://doi.org/10.1046/j.1525-1497.2001.016009606.x

Krupp, L. B., LaRocca, N. G., Muir-Nash, J., & Steinberg, A. D. (1989). The fatigue severity scale. Application to patients with multiple sclerosis and systemic lupus erythematosus. *Arch Neurol*, *46*(10), 1121-1123. https://doi.org/10.1001/archneur.1989.00520460115022

Lee, K. A., Hicks, G., & Nino-Murcia, G. (1991). Validity and reliability of a scale to assess fatigue. *Psychiatry Res*, *36*(3), 291-298. https://doi.org/10.1016/0165-1781(91)90027-m

Lester, D. K., & Zhang, K. (2010). Gait analysis of knee arthritis treated with hyaluronic acid. *J Arthroplasty*, *25*(8), 1290-1294. https://doi.org/10.1016/j.arth.2009.09.001

Matza, L. S., Phillips, G. A., Revicki, D. A., Murray, L., & Malley, K. G. (2011). Development and validation of a patient-report measure of fatigue associated with depression. *J Affect Disord*, *134*(1-3), 294-303. https://doi.org/10.1016/j.jad.2011.06.028

McNair, D. L., M.; Droppleman, L.;. (1992). EDITS manual profile of mood states. *Educational and Industrial Testing Services*.

Mendoza, T. R., Wang, X. S., Cleeland, C. S., Morrissey, M., Johnson, B. A., Wendt, J. K., & Huber, S. L. (1999). The rapid assessment of fatigue severity in cancer patients: use of the Brief Fatigue Inventory. *Cancer*, *85*(5), 1186-1196. https://doi.org/10.1002/(sici)1097-0142(19990301)85:5<1186::aid-cncr24>3.0.co;2-n

Michielsen, H. J., De Vries, J., & Van Heck, G. L. (2003). Psychometric qualities of a brief self-rated fatigue measure: The Fatigue Assessment Scale. *J Psychosom Res*, *54*(4), 345-352. https://doi.org/10.1016/s0022-3999(02)00392-6

Mills, R. J., Young, C. A., Pallant, J. F., & Tennant, A. (2010). Development of a patient reported outcome scale for fatigue in multiple sclerosis: The Neurological Fatigue Index (NFI-MS). *Health Qual Life Outcomes*, *8*, 22. https://doi.org/10.1186/1477-7525-8-22

Nijs, J., Vaes, P., & De Meirleir, K. (2005). The Chronic Fatigue Syndrome Activities and Participation Questionnaire (CFS-APQ): an overview. *Occup Ther Int*, *12*(2), 107-121. https://doi.org/10.1002/oti.19

Okuyama, T., Akechi, T., Kugaya, A., Okamura, H., Shima, Y., Maruguchi, M., Hosaka, T., & Uchitomi, Y. (2000). Development and validation of the cancer fatigue scale: a brief, three-dimensional, self-rating scale for assessment of fatigue in cancer patients. *J Pain Symptom Manage*, *19*(1), 5-14. https://doi.org/10.1016/s0885-3924(99)00138-4

Piper, B. F., Dibble, S. L., Dodd, M. J., Weiss, M. C., Slaughter, R. E., & Paul, S. M. (1998). The revised Piper Fatigue Scale: psychometric evaluation in women with breast cancer. *Oncol Nurs Forum*, *25*(4), 677-684.

Radloff, L. S. (1991). The use of the Center for Epidemiologic Studies Depression Scale in adolescents and young adults. *J Youth Adolesc*, *20*(2), 149-166. https://doi.org/10.1007/BF01537606

Schnelle, J. F., Buchowski, M. S., Ikizler, T. A., Durkin, D. W., Beuscher, L., & Simmons, S. F. (2012). Evaluation of Two Fatigability Severity Measures in Elderly Adults. 1527-1533. https://doi.org/10.1111/j.1532-5415.2012.04062.x

Schwartz, A. L. (1998). The Schwartz Cancer Fatigue Scale: testing reliability and validity. *Oncol Nurs Forum*, *25*(4), 711-717.

Schwartz, J. E., Jandorf, L., & Krupp, L. B. (1993). The measurement of fatigue: a new instrument. *J Psychosom Res*, *37*(7), 753-762. https://doi.org/10.1016/0022-3999(93)90104-n

Schwid, S. R., Thornton, C. A., Pandya, S., Manzur, K. L., Sanjak, M., Petrie, M. D., McDermott, M. P., & Goodman, A. D. (1999). Quantitative assessment of motor fatigue and strength in MS. *Neurology*, *53*(4), 743-750. https://doi.org/10.1212/wnl.53.4.743

Sehle, A., Vieten, M., Sailer, S., Mündermann, A., & Dettmers, C. (2014). Objective assessment of motor fatigue in multiple sclerosis: the Fatigue index Kliniken Schmieder (FKS). *J Neurol*, *261*(9), 1752-1762. https://doi.org/10.1007/s00415-014-7415-7

Shuman-Paretsky, M., Zemon, V., Foley, F. W., & Holtzer, R. (2017). Development and Validation of the State-Trait Inventory of Cognitive Fatigue in Community-Dwelling Older Adults. *Arch Phys Med Rehabil*, *98*(4), 766-773. https://doi.org/10.1016/j.apmr.2016.07.024

Simonsick, E. M., Schrack, J. A., Glynn, N. W., & Ferrucci, L. (2014). Assessing fatigability in mobility-intact older adults. *J Am Geriatr Soc*, *62*(2), 347-351. https://doi.org/10.1111/jgs.12638

Smets, E. M., Garssen, B., Bonke, B., & De Haes, J. C. (1995). The Multidimensional Fatigue Inventory (MFI) psychometric qualities of an instrument to assess fatigue. *Journal of psychosomatic research*, *39*(3), 315-325. https://doi.org/10.1016/0022-3999(94)00125-O

Stein, K. D., Martin, S. C., Hann, D. M., & Jacobsen, P. B. (1998). A multidimensional measure of fatigue for use with cancer patients. *Cancer Pract*, *6*(3), 143-152. https://doi.org/10.1046/j.1523-5394.1998.006003143.x

Tiesinga, L. J., Dassen, T. W., & Halfens, R. J. (1998). DUFS and DEFS: development, reliability and validity of the Dutch Fatigue Scale and the Dutch Exertion Fatigue Scale. *Int J Nurs Stud*, *35*(1-2), 115-123. https://doi.org/10.1016/s0020-7489(98)00005-4

Toosizadeh, N., Joseph, B., Heusser, M. R., Orouji Jokar, T., Mohler, J., Phelan, H. A., & Najafi, B. (2016). Assessing Upper-Extremity Motion: An Innovative, Objective Method to Identify Frailty in Older Bed-Bound Trauma Patients. *Journal of the American College of Surgeons*, *223*(2), 240-248. https://doi.org/10.1016/j.jamcollsurg.2016.03.030

Vercoulen, J. H., Swanink, C. M., Fennis, J. F., Galama, J. M., van der Meer, J. W., & Bleijenberg, G. (1994). Dimensional assessment of chronic fatigue syndrome. *J Psychosom Res*, *38*(5), 383-392. https://doi.org/10.1016/0022-3999(94)90099-x

Visser-Keizer, A. C., Hogenkamp, A., Westerhof-Evers, H. J., Egberink, I. J., & Spikman, J. M. (2015). Dutch multifactor fatigue scale: a new scale to measure the different aspects of fatigue after acquired brain injury. *Arch Phys Med Rehabil*, *96*(6), 1056-1063. https://doi.org/10.1016/j.apmr.2014.12.010

Ware, J., Jr., Kosinski, M., & Keller, S. D. (1996). A 12-Item Short-Form Health Survey: construction of scales and preliminary tests of reliability and validity. *Med Care*, *34*(3), 220-233. https://doi.org/10.1097/00005650-199603000-00003

Ware, J. E., & Gandek, B. (1998). Overview of the SF-36 Health Survey and the International Quality of Life Assessment (IQOLA) Project. *Journal of Clinical Epidemiology*, *51*(11), 903-912. https://doi.org/10.1016/s0895-4356(98)00081-x

Yang, C. M., & Wu, C. H. (2005). The situational fatigue scale: a different approach to measuring fatigue. *Qual Life Res*, *14*(5), 1357-1362. https://doi.org/10.1007/s11136-004-5680-0

Yellen, S. B., Cella, D. F., Webster, K., Blendowski, C., & Kaplan, E. (1997). Measuring fatigue and other anemia-related symptoms with the Functional Assessment of Cancer Therapy (FACT) measurement system. *J Pain Symptom Manage*, *13*(2), 63-74. https://doi.org/10.1016/s0885-3924(96)00274-6

Yesavage, J. A., Brink, T. L., Rose, T. L., Lum, O., Huang, V., Adey, M., & Leirer, V. O. (1982). Development and validation of a geriatric depression screening scale: A preliminary report. *Journal of Psychiatric Research*, *17*(1), 37-49. https://doi.org/10.1016/0022-3956(82)90033-4
